# Tff3 Deficiency Protects against Hepatic Fat Accumulation after Prolonged High-Fat Diet

**DOI:** 10.3390/life12081288

**Published:** 2022-08-22

**Authors:** Kate Šešelja, Iva Bazina, Milka Vrecl, Jessica Welss, Martin Schicht, Martina Mihalj, Vjekoslav Kopačin, Friedrich Paulsen, Tatjana Pirman, Mirela Baus Lončar

**Affiliations:** 1 Divison of Molecular Medicine, Ruđer Boškovic Institute, Bjenicka 54, 10000 Zagreb, Croatia; 2 Institute of Preclinical Sciences, Veterinary Faculty, University of Ljubljana, Gerbiceva 60, 1000 Ljubljana, Slovenia; 3 Institute of Functional and Clinical Anatomy, Faculty of Medicine, Friedrich-Alexander-University Erlangen-Nürnberg, 91054 Erlangen, Germany; 4 Department of Physiology and Immunology, Faculty of Medicine, University of Osijek, J. Huttlera 4, 31000 Osijek, Croatia; 5 Medical Biochemistry Laboratory Kopačin, Prolaz Josipa Leovića 4, 31000 Osijek, Croatia; 6 Department of Animal Science, Biotechnical Faculty, University of Ljubljana, Groblje 3, 1230 Domzale, Slovenia

**Keywords:** trefoil peptide 3, liver, high-fat diet, metabolic syndrome, lipid metabolism

## Abstract

Trefoil factor 3 (Tff3) protein is a small secretory protein expressed on various mucosal surfaces and is involved in proper mucosal function and recovery via various mechanisms, including immune response. However, Tff3 is also found in the bloodstream and in various other tissues, including the liver. Its complete attenuation was observed as the most prominent event in the early phase of diabetes in the polygenic Tally Ho mouse model of diabesity. Since then, its role in metabolic processes has emerged. To elucidate the complex role of Tff3, we used a new Tff3-deficient mouse model without additional metabolically relevant mutations (*Tff3*-/-/C57BL/6NCrl) and exposed it to a high-fat diet (HFD) for a prolonged period (8 months). The effect was observed in male and female mice compared to wild-type (WT) counter groups (*n* = 10 animals per group). We monitored the animals’ general metabolic parameters, liver morphology, ultrastructure and molecular genes in relevant lipid and inflammatory pathways. Tff3-deficient male mice had reduced body weight and better glucose utilization after 17 weeks of HFD, but longer HFD exposure (32 weeks) resulted in no such change. We found a strong reduction in lipid accumulation in male *Tff3*-/-/C57BL/6NCrl mice and a less prominent reduction in female mice. This was associated with downregulated peroxisome proliferator-activated receptor gamma (Pparγ) and upregulated interleukin-6 (Il-6) gene expression, although protein level difference did not reach statistical significance due to higher individual variations. *Tff3*-/-/C57Bl6N mice of both sex had reduced liver steatosis, without major fatty acid content perturbations. Our research shows that Tff3 protein is clearly involved in complex metabolic pathways. Tff3 deficiency in C57Bl6N genetic background caused reduced lipid accumulation in the liver; further research is needed to elucidate its precise role in metabolism-related events.

## 1. Introduction

The liver is a central metabolic organ with a wide variety of roles, such as glucose regulation and lipid metabolism, the proper functioning of which is essential to maintain body homeostasis and overall health [1]. Several conditions can lead to imbalances of lipid metabolism in the liver and abnormal accumulation of triglycerides in hepatocytes, known as steatosis. Obesity-related steatosis or nonalcoholic fatty liver disease (NAFLD) is one of the most prominent hepatic manifestations of metabolic syndrome, closely associated with type 2 diabetes (T2D) [2]. These are major global health problems that have, unfortunately, reached epidemic proportions worldwide [3].

Trefoil factor 3 (Tff3) is a small (6 kDa) secreted protein member of the trefoil factor family (Tff) of proteins, along with Tff1 and Tff2, which are characterized by at least one copy of the trefoil motif, a 40-amino acid domain that contains three conserved disulphides [4]. Tff3 is secretory glycoprotein produced by goblet cells that drives wound healing throughout the gastrointestinal (GI), respiratory, ocular and genitourinary mucosa [5,6]. Tff3 protects injured epithelia by increasing mucus viscosity, facilitating cell migration, inhibiting apoptosis [7] and affecting immune response [8,9]. Less is known about Tff3 function at other sites of expression, which include the brain, pancreas, lymphoid tissue, blood circulation and liver [10]. 

Liver Tff3 has been identified as a novel peptide involved in complex metabolic interactions [11,12,13,14,15,16]. The first association with metabolism emerged from a study of a polygenic diabesity mouse model (Tally Ho) developed by scientists at the Jackson Laboratory, which revealed *Tff3* as the most significantly changed of all the analysed genes. It was transcriptionally active in the livers of control C57BL/6J mice but virtually undetectable in the diabesity model [12]. Downregulation of *Tff3* gene expression in the liver has also been reported in several genetic, as well as diet-induced, mouse models of obesity and diabetes [13,14,15], as well as hepatic steatosis [16]. Overexpression of Tff3 in the liver in the same diabetic and obese mouse models improved the diabetic phenotype [13,14,15]. Moreover, Tff3 was shown to directly bind to the promoter region of peroxisome proliferator-activated receptor alpha (*Pparα*) and upregulate its expression, which subsequently reduced hepatic steatosis by increasing the fatty oxidation process in the liver [15]. In contrast, *Tff3*-deficient (*Tff3-/-*) mice generated on a mixed genetic background (C57BL/6J/Sv129) showed improved utilization of glucose and enhanced insulin sensitivity with increased formation of small lipid vesicles in the liver compared with wild-type (WT) controls but without obvious signs of hepatic steatosis [17]. The only study conducted on patients in the context of diabetes research showed that type 1 diabetes (T1D) patients have decreased serum TFF3 levels compared to healthy controls, which increased after insulin treatment [18]. 

It is evident that Tff3 plays a role in relevant metabolic processes in the liver, although its underlying mechanisms remain unresolved. 

Most biomedical research is conducted on mice colloquially called “Black 6“ (C57Bl6), but many genetic substrains have genetic specificities that contribute to the mouse phenotype [19]. The most common strains, C57BL/6N and C57BL/6J, are derived from the same C57BL/6 parental strain, but they exhibit crucial differences. The C57BL/6 J mouse has a multiexon deletion of the *Nnt* (nicotinamide nucleotide transhydrogenase) gene and shows impaired insulin secretion and glucose homeostasis [20]. Nnt is an important mitochondrial protein that is a major generator of mitochondrial NADPH, regulates cofactor balance and coordinates reductive carboxylation and glucose catabolism in the tricarboxylic acid cycle (TCA) [21]. 

NNT maintains mitochondrial antioxidant capacity through the generation of NADPH [22], and loss of active NNT in 6J mice is associated with reduced ability to detoxify reactive oxygen species (ROS) via the glutathione and thioredoxin pathways [23,24,25]. Because redox regulation is involved in many cellular processes, the loss of Nnt strongly affects various cellular processes and immunological response [26,27,28], resulting in multiple phenotypes [29,30,31]. 

Recently, it was demonstrated that the presence of Nnt and C57BL/6N background, rather than loss of granzyme A expression, in *Gzma*-/- mice was responsible for the phenotype in the viral arthritis model [32], indicating the importance of the currently ignored influence of mouse genetic background [32,33]. Given that humans express functional NNT, these data suggest that the 6N substrain is a more representative model system for studies designed to elucidate the mechanisms by which nutrient excess drives the metabolic syndrome [34].

To determine the impact of Tff3 protein in complex metabolic events, we developed a novel congenic *Tff3-/-* mouse strain on a C57BL/6N genetic background with functional Nnt protein, avoiding the possible contribution of additional mutations present in C57Bl6J and mixed-background strains [35]. Newly generated *Tff3-/-* mice and appropriate WT controls of both sexes were fed a high-fat diet (HFD) for 8 months to induce metabolic syndrome conditions, including fatty liver phenotype.

Considering the observed differences in liver Tff3 expression in males and females [11] and the growing awareness of the need to include both sexes in biomedical research on various human diseases [36], we additionally analysed the contribution of sex to the effects of Tff3 deficiency on the metabolic parameters. We monitored weight, glucose and insulin tolerance during the experiment. General blood biochemical markers were determined, and fatty acid content, morphology and the ultrastructure of livers were examined. We monitored the expression of genes involved in liver steatosis pathology-related pathways of fatty acid metabolism and inflammation. 

## 2. Materials and Methods

### 2.1. Animals and Diet Treatment

A trefoil factor family 3 (*Tff3*)-deficient mouse strain on a C57BL/6NCrl (Charles River) genetic background was developed from an existing mixed-background strain (C57BL/6J/SV129) using a ‘speed congenics’ approach as described previously [13]. *Tff3*-deficient mice (*Tff3-/-*/C57BL/6NCrl) and appropriate wild-type strain C57BL6/NCrl controls were raised in the facility for laboratory animals of the Ruđer Bošković Institute under standard care conditions. Female and male mice of wild-type (C57BL/6NCrl) and Tff3-deficient genotype (*Tff3*-/-/C57Bl/6NCrl) were monitored. Animals were fed an HFD (Ssniff, E15742-34, 24.4% crude protein, 34.6% crude fat, 6.0% crude fiber, 5.5% crude ash, 0.1% starch and 9.4% sugar) from weaning until they were sacrificed 8 months later. Ten animals per group (five of which were used for fixation by total body fixative perfusion and five for fresh tissue collection) were weighed, and blood glucose levels were determined at the ages of 21 and 36 weeks. All measurements were carried out after mice fasted for 16 h, specifically from 6 p.m. to 10 a.m. In addition, animals that were used as controls in weight measurement experiments were fed a standard diet (Mucedola, 4RF21). Mice were kept at 21 °C with 60% humidity and a 12 h light–dark cycle. Experimental animal manipulations and procedures performed during the study under the Croatian Science Foundation grant IP-06-2016-2717 were approved by the local ethical committee.

### 2.2. Glucose and Insulin Tolerance Test

Intraperitoneal glucose tolerance tests (IPGTTs) were performed on animals at 21 (17 weeks of HFD) and 36 weeks of age (32 weeks of HFD). The tests were performed according to the International Mouse Phenotyping Resource of Standardised Screens protocol [37]. After a 16 h fasting period, 2 g/kg glucose in sterile 1xPBS was administered intraperitoneally. The blood glucose level was measured at the beginning of experiment and at 15, 30, 60 and 120 min after glucose injection from a tail vein. Water was available ad libitum. 

Intraperitoneal insulin tolerance tests (IPITTs) were performed on animals at 23 (19 weeks of HFD) and 38 weeks of age (34 weeks of HFD). The tests followed the Mouse Metabolic Phenotyping Centers protocol [38]. Animals were fasted for 4 h with water available ad libitum. Whole blood glucose level was measured from the tip of the tail before intraperitoneal application of 0.75IU/kg of insulin and 15, 30, 45, 60 and 120 min post injection. 

### 2.3. Histological Analysis

Livers were fixed in 10% buffered formalin (Shandon Formal-Fixx 10% neutral buffered formalin; Thermo Scientific GmbH, Vienna, Austria). After fixation, samples were dehydrated and embedded in paraffin blocks (Tissue-Tek^®^ TEC™ 5 Tissue Embedding Console System, Sakura Finetek Europe B.V.; Alphen aan den Rijn, The Nederlands) according to a standard procedure. Next, 5 µm histological sections were cut using a Leica SM 2000R microtome (Leica Biosystems, Nussloch, Germany), stained with haematoxylin and eosin (HE) and a Masson–Goldner kit (Merck SA, Darmstadt, Germany) and cover-slipped using a Gemini AS automated slide stainer and a ClearVue cover slipper (Thermo Fisher Scientific, Waltham, MA, USA). Oil Red O staining was performed to visualize lipid accumulation. Formalin-fixed liver samples were frozen in liquid nitrogen, and 10 μm cryosections were cut using a Leica CM 1800 cryostat (Leica Biosystems, Nussloch, Germany) at −17 °C, mounted on Thermo Scientific™ Superfrost^®^ Plus slides (Gerhard Menzel B.V. & Co. KG, Braunschweig, Germany) and stored at −80 °C. The thawed and air-dried cryosections of the liver samples were then rinsed with deionized water and stained with Oil Red O (Merck SA, Darmstadt, Germany) staining solution (0.5% Oil Red O in isopropanol) for 10 min, and the cell nuclei were counterstained with haematoxylin. After rinsing with tap water, sections were mounted with aqueous mounting medium (Aquatex^®^; Merck SA, Darmstadt, Germany). A Nikon Microphot FXA microscope with a DS-Fi1 camera and NIS Elements BR 4.6 imaging software (Nikon instruments Europe B.V., Badhoevedorp, The Netherlands) was used for histological examination. Representative tissue sections were presented using Adobe Creative Cloud.

### 2.4. Ultrastructural Analysis

Mice of both genotypes and sexes were perfused with 4% PFA. Tissues were immersion-fixed in Ito’s fixative immediately after collection. Liver samples were postfixed in OsO4 and dehydrated in a graded ethanol series, and the tissues were embedded in Epon resin. Tissue sections (1 µm thick) were cut with an ultramicrotome (Ultracut E; Reichert Jung, Vienna, Austria) and stained with toluidine blue. Toluidine-blue-stained slides were examined with a Biorevo BZ-9000 microscope (Keyence, Neu-Isenburg, Germany). Ultrathin sections of the tissue were cut, stained with uranyl acetate and lead citrate and inspected with a JEM-1400Plus transmission electron microscope (JEOL (Germany) GmbH, Freising, Germany).

### 2.5. Q-PCR Analysis

Liver tissues were collected, snap-frozen in liquid nitrogen and stored at −80 °C for further analysis. Total RNA was isolated from the livers of *Tff3-/-* and WT mice of both sexes using a NucleoSpin RNA (MACHEREY-NAGEL, Düren, Germany) kit according to the manufacturer’s protocol. RNA was transcribed into cDNA with a high-capacity cDNA reverse transcription kit (Applied Biosystems, Dreieich, Germany). Quantitative polymerase chain reaction (qPCR) was performed using SYBR Green I (Invitrogen, Waltham, MA, USA) detection chemistry and specific primers (Table S1) on a StepOnePlus™ qPCR system (Applied Biosystems). The cycling conditions were as follows: three minutes of polymerase activation at 95 °C and 40 cycles comprising 95 °C for 1min, annealing temperature specific for each primer pair (Table S1) for 30 s and elongation at 72 °C for 30 s. A single product amplification was confirmed by melting curve analysis and polyacrylamide gel electrophoresis. Gene expression was normalized to stable housekeeping genes, β-actin (*Actβ*) and β2-microglobulin (β*2m*). Changes were represented as fold change.

### 2.6. Western Blot

Total liver proteins were isolated from *Tff3-/-* and WT mice fed with an HFD for 8 months (*n* = 5 animals per group). RIPA buffer (50 mM TRIS HCL, pH8, 150 mM NaCl, 1 mM EDTA, 1% NP40, 1% sodium deoxycholate, 0.1% SDS) supplemented with phosphatase and protease inhibitors was used for isolation. Protein concentration was determined by a BCA protein assay kit (Pierce, Thermo Fischer, Waltham, MA, USA), and 10 µg of proteins per lane was separated by sodium dodecyl sulphate-polyacrylamide gel electrophoresis (SDS-PAGE). Proteins were transferred to a PVDF membrane overnight at the constant 100 mA. I-Block™ protein-based blocking reagent (T2015) was used for blocking for 1h. Il-6 primary antibody (ab208113) and Pparγ primary antibody (sc7196) were incubated overnight at 4 °C with dilutions of 1:1000. Goat anti-rabbit IgG-HRP (#170-6515; Bio-rad) was used for detection. The chemiluminescence signals were detected (Uvitec Alliance Q9 mini) and analysed with Image J (version 1.53s, National Institute of Health, Bethesda, MD, USA). Bands were normalized by amido black stain.

### 2.7. Fatty Acid Analyses

Fatty acid composition of the liver was analyzed using gas chromatography. The procedure can be briefly summarized as follows: 0.25 g of homogenised sample was transmethylated in situ according to the method of Park and Goins [39] using 0.5 M NaOH in methanol, followed by 14% BF3 in methanol. Fatty acid methyl esters (FAME) were extracted using hexane. For FAME separation, an Agilent 6890 GC equipped with a DB-Fatwax UI chromatographic column (30 m length; 0.25 mm i.d., 0.25 m film thickness; Agilent Technologies, Santa Clara, CA, USA) and FID detector was used. The results are expressed as weight percentage (g of individual FA per 100 g of total FA) or in g/kg of sample (calculated using weight percentage of FAs and fat content in the sample).

### 2.8. Statistical Analyses

Body weight measurements and results from IPGTT and IPITT were analysed by two-way ANOVA, followed by a Tukey post hoc test. Blood sera were analysed using two-way ANOVA, followed by a Bonferroni post hoc test. Liver fat and fatty acid content were analysed using general linear model (GLM) procedures of the SAS/STAT module (SAS Institute Inc., Cary, NC, USA), with the differences determined by a Tukey–Kramer multiple comparison test, taking into consideration the genotype as the main effect, separately for male and female mice. Gene expression was analyzed by REST © software (ΔΔCt method) and normalized to stable housekeeping genes, β-actin (*Actβ*) and β2-microglobulin (β*2m*). Changes were represented as fold change. All graphs were generated using GraphPad Prism version 8.0.0 (GraphPad Software, San Diego, CA, USA).

## 3. Results

### 3.1. General Metabolic Parameters (Weight Measurments and Glucose and Insulin Tolerance Test)

**Figure 1:**
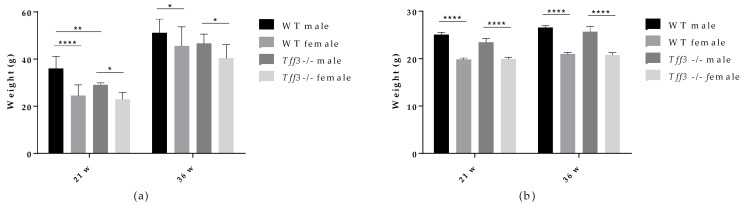
Effect of long-term HFD treatment on body weight in WT and Tff3-deficient mice of both sexes and age-matched controls fed a standard diet (*n* = 10 animals per group). (**a**) Body weight of 21-week- (21 w) and 36-week (36 w)-old mice fed an HFD from weaning; (**b**) body weight of 21-week- (21 w) and 36-week (36 w)-old mice fed an SD; * *p* ≤ 0.05, ** *p* ≤ 0.01, **** *p* ≤ 0.0001.

Body weight was determined in WT and *Tff3-/-* mice of both sexes fed an HFD (Figure 1a) and in age-matched control mice fed a standard diet (SD) (Figure 1b). Mice at a given age (21 and 36 weeks) were weighed after 16 h of fasting. As expected, female mice (HFD- and SD-fed) weighed less than male mice at both time points (Figure 1a,b). HFD-fed male *Tff3-/-* mice (21 weeks old; 17 weeks HFD) had significantly lower body weights than WT male mice (Figure 1a). This difference was lost after prolonged exposure to an HFD (36 weeks old; 32 weeks HFD), when the body weights of WT and male *Tff3-/-* mice were similar (Figure 1a). There were no genotype-related differences in body weight in mice fed standard diets (Figure 1b). This suggests that Tff3 deficiency does not cause a difference in feeding habits or energy intake. Because we were interested in elucidating the effect of Tff3 deficiency in the case of metabolic syndrome conditions and liver steatosis, we further concentrated on the effects of HFD exposure in WT and *Tff3-/-* mice of both sexes. 

**Figure 2:**
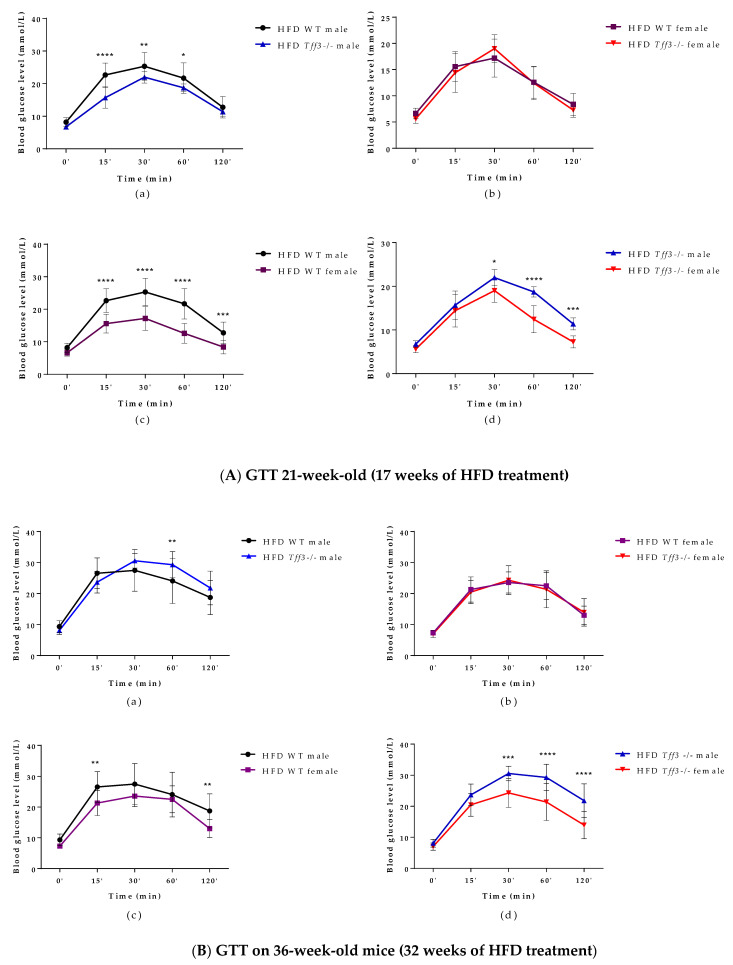
Intraperitoneal glucose tolerance test (GTT) performed on 21- (**A**) and 36-week-old (**B**) (17 and 32 weeks of HFD treatment) WT and *Tff3-/-* mice (male and female). Blood glucose levels were measured at time points 0, 15, 30, 60 and 120 min after glucose injection (2 mg/g body mass) and are presented as (**a**) WT male compared to *Tff3-/-* male, (**b**) WT female compared to *Tff3-/-* female, (**c**) WT male compared to WT female and (**d**) *Tff3-/-* male compared to *Tff3-/-* female; two-way ANOVA (Tukey post hoc test) was used for statistical analysis, and significant time points are marked as * *p* ≤ 0.05; ** *p* ≤ 0.01; *** *p* ≤ 0.001; **** *p* ≤ 0.0001.

An intraperitoneal glucose tolerance test (GTT) was performed on 21-week-old (17 weeks of HFD treatment) WT and *Tff3-/-* mice (male and female) (Figure 2A). *Tff3*-/- male mice showed improved glucose tolerance compared to WT male mice at 15, 30 and 60 min after glucose administration (Figure 2(Aa)), whereas *Tff3-/-* female mice did not show any statistically relevant differences compared to WT females (Figure 2(Ab)). WT male mice had impaired glucose tolerance compared to WT female mice at almost every time point (Figure 2(Ac)). *Tff3-/-* male mice showed impaired glucose tolerance compared to *Tff3-/-* female mice at 30, 60 and 120 min post glucose administration (Figure 2(Ad)). 

To further assess glucose homeostasis, GTT was also performed at the end of experiment (32 weeks of HFD treatment) on 36-week-old WT and *Tff3-/-* mice (male and female) (Figure 2B). *Tff3-/-* male mice showed impaired glucose tolerance when compared to WT male mice, but only at 60 min after glucose administration (Figure 2(Ba)). Differences were also less obvious when comparing WT male to WT female mice (Figure 2(Bc)), but *Tff3-/-* male mice clearly showed worsened glucose tolerance when compared to *Tff3-/-* female mice (Figure 2(Bd)).

**Figure 3:**
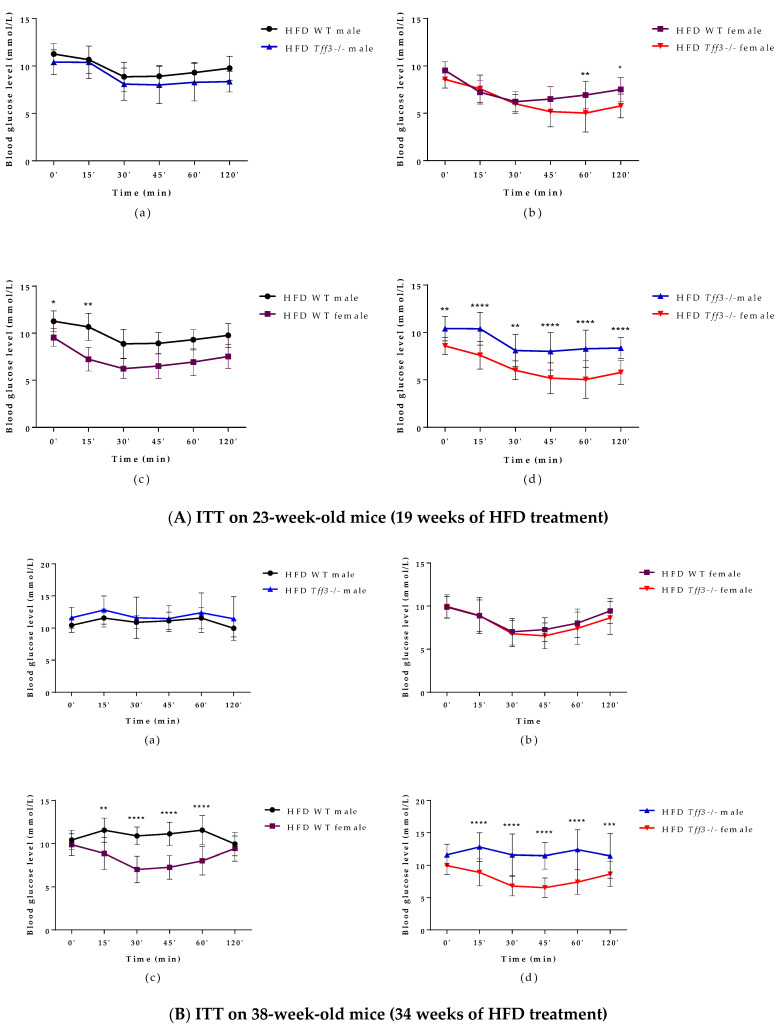
Intraperitoneal insulin tolerance test (ITT) performed on 23- and 38-week-old (19 and 34 weeks of HFD treatment) WT and *Tff3 -/-* mice (male and female). Blood glucose levels were measured at time points 0, 15, 30, 45, 60 and 120 min after glucose injection (2 mg/g body mass) and presented as (**a**) WT male compared to *Tff3-/-* male, (**b**) WT female compared to *Tff3-/-* female, (**c**) WT male compared to WT female and (**d**) *Tff3-/-* male compared to *Tff3-/-* female; two-way ANOVA (Tukey post hoc test) was used for statistical analysis, and significant time points are marked as * *p* ≤ 0.05; ** *p* ≤ 0.01; *** *p* ≤ 0.001; **** *p* ≤0.0001.

An intraperitoneal insulin tolerance test (ITT) was performed on 23-week-old (Figure 3A) (19 weeks of HFD treatment) and 38-week-old (Figure 3B) (34 weeks of HFD treatment) WT and *Tff3-/-* mice of both sexes. The only difference concerning genotype was that *Tff3-/-* female mice had improved insulin tolerance 60 and 120 min after treatment when compared to WT female mice (Figure 3(Ab)), but when ITT was performed on 38-week-old-animals, this difference diminished (Figure 3(Bb)). WT and *Tff3-/-* male mice showed significantly worsened insulin tolerance in both ITT tests when compared to female mice of same genotype (Figure 3(Ac,Ad,Bc,Bd)).

Metabolic tests of GTT and ITT were also performed on comparable age/sex and genotype of mice fed a standard diet, showing different dynamics of response and glucose utilization (Figures S3 and S4). 

### 3.2. Blood Serum Biochemistry

Biochemical serum analysis was performed at the end of the experiment (9-month-old mice; 8 months on HFD) to assess general health status (Table 1). A significant sex × genotype interaction was found only in the case of ALP, whereby WT male mice presented with significantly higher levels compared to *Tff3-/-* male mice (81.8 ± 5.86 vs. 51.0 ± 7.57; *p* = 0.031). Levels of low-density lipoprotein (LDL), high-density lipoprotein, total cholesterol, triglycerides, blood glucose (BG), aspartate aminotransferase (AST), alanine aminotransferase, C-reactive protein (CRP), urea and total protein levels were not significantly changed in the context of genotype × sex.

### 3.3. Liver Morphology and Ultrastructure

**Figure 4:**
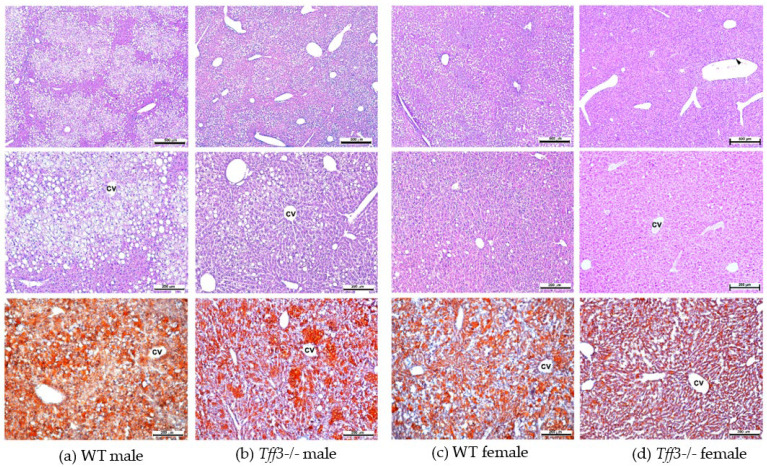
Histologic evaluation of livers from male and female 40-week-old WT and *Tff3-/-* mice. Representative liver sections were stained with H&E (upper and middle row panels) and Oil Red O (bottom row panels). Scale bars = 500 µm (upper row panels) and 200 µm (middle and bottom row panels). H&E—haematoxylin and eosin, cv—central vein; arrowhead—inflammatory cell infiltration (marked in column d).

**Figure 5:**
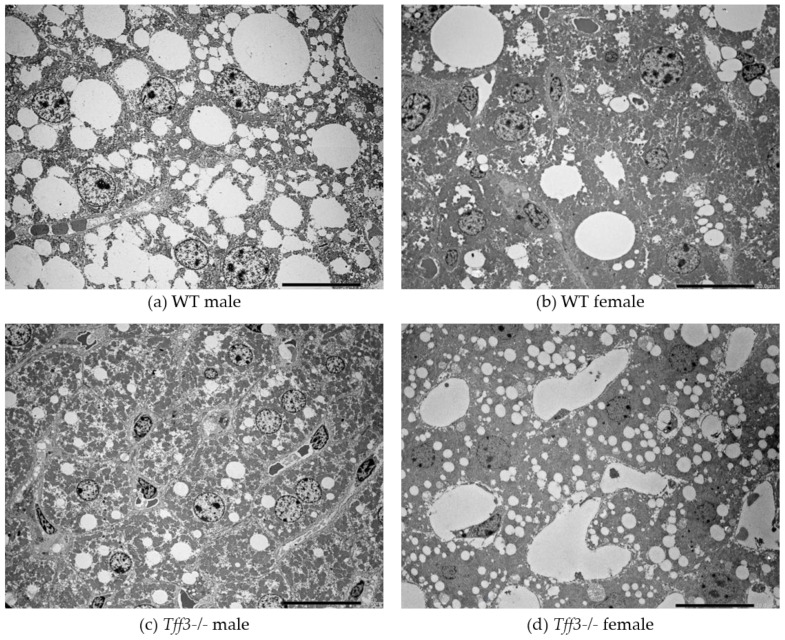
TEM images of the liver of 40-week-old WT mice vs. *Tff3-/-* mice. scale bar = 20 µm.

Histological assessment of liver sections by light microscopy revealed sex- and genotype-related effects on liver histomorphology (Figure 4). Signs of hepatic steatosis in the pericentral (zone 3) and midzone (zone 2) region of the liver lobule were most pronounced in WT males. In haematoxylin and eosin (HE)-stained sections, numerous hepatocytes with large single or smaller unstained vacuoles in the cytoplasm and nuclei located at the periphery were visible, which is characteristic of macrovesicular steatosis. Accumulation of neutral lipids in the liver was also confirmed by Oil Red O staining of liver cryosections (Figure 4). In *Tff3-/-* mice, a marked reduction in hepatic steatosis was observed, especially in *Tff3-/-* males. In female mice, the signs of steatosis were milder compared to those in male mice. In WT female mice, hepatocytes with macrovesicular steatosis were also present and were more numerous than in *Tff3-/-* female mice (Figure 5). Ultrastructural analysis also confirmed that the size of lipid droplets in *Tff3-/-* male mice was smaller. *Tff3-/-* female mice appeared to have an increased number of lipid droplets of the same size (Figure 5). Severity of steatosis has been reported to be positively associated with pericentral (zone 3) fibrosis and lobular inflammation [40]. Masson–Goldner trichrome staining was used to visualize the extent of fibrogenesis but showed no obvious signs of pericellular fibrosis (Figure S1), an observation that is also consistent with ultrastructural analysis. Inflammatory cell infiltrations were noted in individual liver sections but were not specific to individual groups of mice (Figure S1).

In addition, we stained liver sections from control animals fed an SD with H&E and Oil-red O (Figure S5). Sex- and genotype-related effects on liver histomorphology were not observed in liver sections stained with H&E (Figure S3). However, Oil-red O staining of liver cryosections showed a smaller size of lipid droplets in the hepatocytes of Tff3-/- mice compared with WT mice when fed an SD (Figure S5).

### 3.4. Average Fatty Acid Content in Liver and Abdominal Fat

We determined specific content of main fatty acids (g/100 g of total fatty acids) in the liver (Table 2) and abdominal fat (Table S2) of HFD-fed *Tff3-/-* and WT animals. *Tff3-/-* mice *(*male and female) on an HFD had significantly reduced total liver fat content (almost twofold) compared to WT mice (Table 2). We found no relevant genotype difference in specific fatty acid content per 100 g of total fatty acids. Several fatty acids differed between sexes of both WT and *Tff3-/-* animals. The content of main fatty acids in abdominal fat of animals fed an HFD showed no significant genotype differences (Table S2).

Interestingly, *Tff3-/-* mice of both sexes had significantly reduced liver fat content, even when fed an SD (Table S3). In contrast to the HFD group, we found some genotype differences in several major fatty acids in the SD group (Table S3).

**Table 2:** Liver fat (average ± standard error) and fatty acid content (average) in mice on a high-fat diet.

Main Fatty Acids		Group		
(g of Fatty Acids/100 g of Total Fatty Acids)	WT ♂	*Tff3-/-* ♂	WT ♀	*Tff3-/-* ♀
C 14:0	0.47	0.45	0.44	0.45
C 16:0	24.74	24.84	24.18	24.25
C 16:1	5.26	4.46	3.74	3.22
C 18:0	3.51	4.69	5.53	6.83
C 18:1	43.04	38.00	41.16	33.19
C 18:2, n-6	11.40	12.98	12.38	14.72
C 18:3, n-6	0.28	0.30	0.37	0.51
C 18:3, n-3	0.27	0.34	0.32	0.47
C 20:1, n-9	1.05 *	0.86 †	0.54	0.33
C 20:3, n-6	0.74 *	0.78 †	0.54	0.33
C 20:4, n-6	3.72 *	5.26 †	5.13	7.21
C 20:5, n-3	0.17	0.25	0.22	0.34
C 22:4, n-6	0.48	0.51 †	0.30	0.43
C 22:5, n-6	0.23	0.25 †	0.15	0.24
C 22:5, n-3	0.41 *	0.50 †	0.29	0.45
C 22:6, n-3	2.95 *	4.17	3.91	5.78
∑ SFA ^1^	29.28	30.63	30.58	32.07
∑ MUFA ^2^	49.75	43.68	45.70	37.00
∑ PUFA ^3^	20.49	25.18	23.43	30.51
n-6/n-3 PUFA ^4^	4.59:1	3.92:1	4.26:1	3.28:1
Fat content (g/100 g liver)	22.17 ± 1.18 ‡	14.37 ± 1.67	17.21 ± 1.87 §	8.64 ± 1.25

^1^ Saturated fatty acids. ^2^ Monounsaturated fatty acids. ^3^ Polyunsaturated fatty acids. ^4^ Ratio of omega-6 to omega-3 polyunsaturated fatty acids. Results are presented as mean and mean *±* SD (for fat content) and were analysed using general linear model (GLM) procedures of the SAS/STAT module (SAS Institute Inc., Cary, NC, USA), with the differences determined by a Tukey–Kramer multiple comparison test, taking into consideration the genotype as the main effect, separately for male and female mice. Statistical significance was considered at *p* ≤ 0.05.—* WT ♂ vs. WT ♀ (sex-related diff.). †—Tff3-/-o ♂ vs. Tff3-/- ♀ (sex-related diff.). ‡—WT♂ vs. Tff3-/- ♂ (gene-related diff.). §—WT ♀ vs. Tff3-/- ♀ (gene-related diff.).

### 3.5. Expression of Tff3, Fatty Acid Metabolism and Inflammation-Related Genes upon HFD Exposure

**Figure 6:**
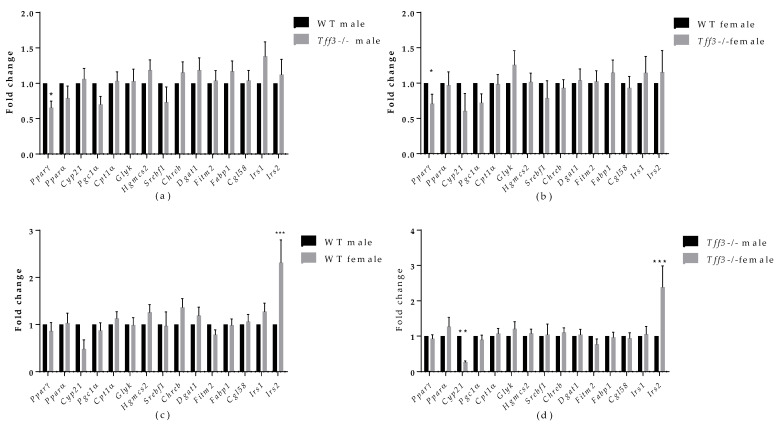
Effect of long-term treatment with HFD on the expression of markers of fatty acid metabolism in the livers of male and female WT and *Tff3-/-* mice. We performed qPCR for all animal groups *(n* = 5) using a SYBR green detection system. Ct values were analysed using REST © software, and results are expressed as fold change. Gene expression in *Tff3-/-* male (**a**) and *Tff3-/-* female (**b**) mice is presented relative to their WT counterparts. Gene expression in WT female (**c**) and *Tff3-/-* female (**d**) mice is presented relative to the corresponding male mice. * *p* ≤ 0.05, ** *p* ≤ 0.01, *** *p ≤* 0.001. *Pparγ*—peroxisome proliferator-activated receptor gamma; *Pparα*—peroxisome proliferator-activated receptor alpha; *Cyp21*—cytochrome P450, family 21, subfamily a, polypeptide 1; *Pgc1α*—peroxisome proliferator-activated receptor-gamma coactivator 1 alpha; *Cpt1α*—carnitine palmitoyltransferase I; *Glyk*—glycerol kinase; *Hgmcs2*—3-hydroxy-3-methylglutaryl-CoA synthase 2; *Srebf1*—sterol regulatory element-binding transcription factor 1; *Chreb*—carbohydrate response element-binding protein; *Dgat1*—diacylglycerol O-acyltransferase 1; *Fitm2*—fat storage-inducing transmembrane protein 2; *Fabp1*—fatty acid-binding protein 1; *Cgl58*—alpha/beta-hydrolase domain containing 5; *Irs1*—insulin receptor substrate 1; *Irs2*—insulin receptor substrate 2.

**Figure 7:**
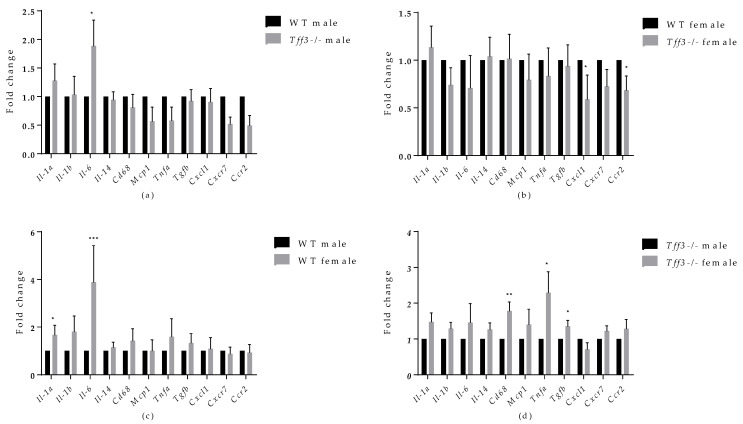
Effect of long-term treatment with HFD on the expression of markers of inflammation in the livers of male and female WT and *Tff3-/-* mice. We performed qPCR for all animal groups (*n* = 5) using a SYBR Green detection system. Ct values were analysed using REST © software, and results are expressed as fold change. Gene expression in *Tff3-/-* male (**a**) and *Tff3-/-* female (**b**) mice is presented relative to their WT counterparts. Gene expression in WT female (**c**) and *Tff3-/-* female (**d**) mice is presented relative to the corresponding male mice. * *p* ≤ 0.05, ** *p* ≤ 0.01; *** *p ≤* 0.001; *Il-1α*—interleukin 1 alpha*; Il-1β*—interleukin 1 beta; *Il-6*—interleukin 6; *Il-14*—interleukin 14; *Cd68*—mouse CD68 antigen; *Mcp1*; *Tnfα*—tumour necrosis factor alpha, *Tgfβ*—tumour growth factor beta; *Cxcl1*— C-X-C motif chemokine ligand 1; *Cxcr7*—atypical chemokine receptor 3; *Ccr2*—chemokine (C-C motif) receptor 2.

We monitored various genes involved in fatty acid metabolism (Figure 6) and inflammation (Figure 7). Regarding fatty acid metabolism (Figure 6a), genotype comparison showed that *Tff3-/-* male mice had statistically relevantly downregulated expression of peroxisome proliferator-activated receptor gamma *(Pparγ)* compared with male WT mice fed an HFD for a period of 8 months. Moreover, *Pparγ* was reduced in *Tff3-/-* female mice compared with WT female mice (Figure 6b). As for the differences in gene expression between the sexes, both WT females and *Tff3-/-* females showed a significant increase in insulin receptor substrate 2 (*Irs2)* compared with WT males and *Tff3-/-* males, respectively (Figure 6c,d). Additionally, *Tff3-/-* males show statistically relevant downregulation of cytochrome P450, family 21, subfamily a, polypeptide (*Cyp21*) gene expression (Figure 6d).

Analyses of inflammatory markers showed that *Tff3-/-* male mice exhibited significant upregulation of *Il-6* compared with WT male mice (Figure 7a). In addition, *Tff3-/-* female mice showed significant downregulation of C-X-C motif chemokine ligand 1 (*Cxcl1)* and chemokine (C-C motif) receptor 2 (*Ccr2)* compared with WT female mice (Figure 7b). 

When comparing the sex effect on specific inflammatory markers (Figure 7c,d), WT female mice exhibited statistically significant upregulation of *Il1α* and *Il-6* compared with WT male mice, whereas *Tff3-/-* female mice exhibited significant upregulation of mouse CD68 antigen (*Cd68)*, tumour necrosis factor alpha (*Tnfα)*, and tumour growth factor beta (*Tgfβ)* compared with *Tff3*-/- male mice. 

Furthermore, we were interested in testing whether there was a sex difference in liver *Tff3* gene expression and whether this expression changed with HFD treatment. Therefore, we monitored gene expression of *Tff3* in WT animals of both sexes in the HFD model and compared the levels with age-matched WT controls on an SD (Figure S2). HFD exposure, compared to SD, strongly reduced liver Tff3 expression in male but not in female mice (Figure S2a,b). Female mice had considerably reduced Tff3 gene expression in the liver compared with male mice under both SD and HFD (Figure S2c,d).

qPCR analyses did not show any differences in SD-fed animals regarding genes that were significantly changed between WT and *Tff3-/-* animals fed an HFD (Figure S6). 

### 3.6. Protein Accumulation of Il-6 and Pparγ in Liver of Tff3-/- and WT Male Mice Fed an HFD for 8 Months

**Figure 8:**
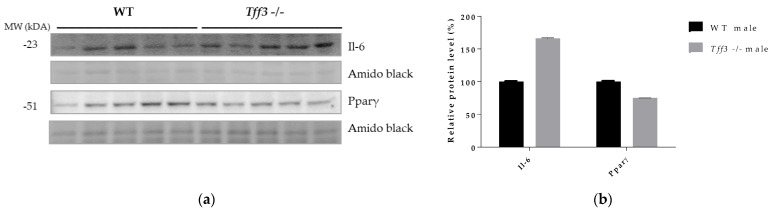
Effect of long-term treatment with HFD on Il-6 and Pparγ protein expression in liver tissue of male WT and *Tff3-/-* mice. (**a**) SDS-PAGE analysis of liver tissue of wild-type male C57BL/6N mice and male *Tff3-/-* mice fed an HFD for 36 weeks, immunoblotted with antibodies for Il-6 and Ppar*γ*; amido black staining is shown as a loading control. (**b**) ImageJ was used for densitometry analysis, and quantification is presented as relative amounts of the protein of interest normalized against amido black signal. Excel’s T test was used for statistical analysis.

Protein expression of IL-6 and Pparγ in liver tissue of WT and Tff3-/- male mice fed an HFD for 36 weeks was detected by Western blot. The results show the same trend that we observed at the level of gene expression but without statistical significance. Il-6 was upregulated and Pparγ was downregulated in male *Tff3-/-* mice compared with WT male mice (Figure 8).

## 4. Discussion

The aim of the present study was to investigate, for the first time, the effects of Tff3 deficiency in a C57Bl6N genetic background on the general metabolic status of animals, focusing on the phenotype of the liver in a model of prolonged fat overload. Therefore, 8 months (35 weeks) of HFD treatment was used to induce the metabolic syndrome in male and female congenic *Tff3-/-* mice and corresponding WT controls (C57Bl6N). We chose chronic exposure to mimic the lifelong dietary pattern in humans and to analyse the effects of Tff3 deficiency on metabolic parameters, focusing on liver health and mechanisms underlying NAFLD, such as inflammation and fatty acid metabolism. [41,42]. To account for any metabolic effects of Tff3 deficiency itself, we also fed a group of age- and sex-matched mice a standard diet. WT and *Tff3-/-* mice of both sexes fed an SD showed no statistical differences in body weight at any given age (21 and 36 weeks) (Figure 1b). Interestingly, *Tff3-/-* mice on mixed genetic background, as we have previously reported, had significantly lower body weight than WT mice after 5 months of SD [43]. This discrepancy could be explained by the impact of a mixed background with unknown genetic inputs, whereas we used a new congenic *Tff3-/-* strain on a clean C57BL/6N genetic background without additional metabolically relevant mutations.

In contrast to the results observed in SD mice, male *Tff3-/-* mice had a statistically significant lower body weight than male WT mice after 17 weeks of HFD (at 21 weeks of age) (Figure 1a). At 36 weeks of age and after 32 weeks of HFD exposure, male *Tff3-/-* mice still weighed less than male WT mice, but this trend appeared to attenuate with treatment duration (Figure 1a). Thus, it appears that the weight differences between male *Tff3-/-* and WT mice were less pronounced after longer HFD exposure (32 weeks). This was also the case when we analysed overall glucose homeostasis and insulin sensitivity. We performed glucose and insulin tolerance tests at the same time points (21 and 36 weeks of age), waiting two weeks between experiments to allow the mice the necessary recovery time (Figure 2). *Tff3-/-* male mice showed better glucose utilization compared to WT mice at 15, 30 and 60 min after glucose administration, but this was only the case when we performed the test at 21 weeks of age (Figure 2a). The difference diminished when we examined the same animals later, at 32 weeks of age (Figure 2(Ba)). Standard-diet-fed male mice of the same age showed no difference at 21 weeks of age, but at 36 weeks, Tff3-deficient males were more efficiently removing glucose from circulation (Figure S3(Ba)). Our previous observations on *Tff3-/-* mice (21 weeks old) on a mixed background fed an SD showed better glucose utilization at 15 and 30 min after administration [17]. In contrast, overexpression of Tff3 improved glucose tolerance in B6D2F1 mice fed an HFD for 6 weeks [12], in male Lepr db/Leprdb (db/db) mice and Leprob/Leprob (ob/ob) and in the diet-induced obesity mouse model (8-week-old C57BL/6 mice fed an HFD for more than 8 weeks) [13]. It is difficult to compare results between studies because there are many parameters that can affect the test outcome, e.g., fasting time before glucose administration, different mouse strains, different compositions and duration of the high-fat diet and different time points. Additionally, considering the difference in genetic environment of these mouse models, these differences are not surprising. 

As for insulin tolerance tests in the HFD model, (Figure 3), there was no major difference in insulin sensitivity due to *Tff3* deficiency alone, with the exception of *Tff3*-/- females, which showed higher sensitivity compared to WT females at the end of the test (60 and 120 min) (Figure 3(Ab)). This difference diminished in the test performed on animals at 38 weeks of age (Figure 3(Bb)). Consistent with existing data, our results with respect to glucose and insulin tolerance tests and weight measurements suggest that WT female mice are partially protected from HFD treatment compared to WT males (Figure 1a, Figure 2(Ac,Bc) and Figure 3(Ac,Bc)) [44]. Clearly, HFD affects the phenotype in a sexually dimorphic manner, but the exact reasons remain unknown [45,46,47,48]. This phenomenon of a stronger male effect in response to HFD treatment also applied to the *Tff3-/-* animals. HFD-fed Tff3-deficient males had worsened glucose and insulin tolerance at almost all time points compared with the *Tff3-/-* female mice (Figure 2(Ad,Bd) and Figure 3(Ad,Bd)). We could not find any publications addressing the role of sex variables regarding Tff3 and HFD, so to the best of our knowledge, this is the first study to report such results. Interestingly, sex-related differences in *Tff3* gene expression in the livers of adult C57BL/6J mice on an SD were reported. Specifically, *Tff3* expression in male livers was higher than *Tff3* expression in female livers [11]. We observed the same phenomenon in our WT (C57BL/6N) mouse models in both SD-fed mice (Figure S2c) and HFD-fed mice (Figure S2d). In addition, we analysed the effects of HFD on Tff3 expression in the liver of male and female C57BL6N mice compared with the SD expression level (Figure S2a,b). A high-fat diet resulted in decreased expression of the Tff3 gene in male mice (Figure S2a), confirming the results of previous studies [12,13,14,15]. Interestingly, female C57B76N mice had the same level of Tff3 gene expression in the liver in the SD and HFD models (Figure S2b). Functional assays (GTT and ITT) in SD-fed mice (Figures S3 and S4) at the same age as the HFD groups show that Tff3 deficiency does not affect male response to glucose overload until 36 weeks of age (Figure S3) and to insulin overload until 21 weeks of age (Figure S4). Female mice deficient in Tff3 exhibited better glucose utilization at one time point (30 min) at 21 weeks of age. These data reflect the different dynamics of glucose utilization with aging on a standard diet.

Lipid homeostasis in the liver illustrates the complexity of metabolic regulation, and its imbalance can lead to a variety of metabolic syndrome disorders, including insulin resistance, T2D and NAFLD. A major pathological feature of these complex manifestations of metabolic syndrome is the aberrant accumulation of lipid droplets in hepatocytes, or hepatic steatosis [2]. A role of Tff3 in the metabolism of fatty acids in the liver was previously described [15,17]; therefore, we examined the effects of long-term HFD on our *Tff3-/-*C57Bl6/NCrl mouse strain. 

Histological analyses revealed that *Tff3-/-* C57Bl6N mice exhibit a decrease in the size of lipid droplets compared with WT mice (Figure 4). Interestingly, data from the SD-fed control group showed the same trend in lipid droplet size, although on a much smaller scale (Figure S5), which indicates that Tff3 is somehow involved in lipid metabolism in the liver. Hepatic sexual dimorphism is present in health and disease [49,50,51,52,53,54,55], so the difference between sexes in not surprising. Males of both genotypes have larger lipid droplets on both feeding treatments (Figure 4 and Figure S5). The size of lipid droplets, as confirmed by ultrastructrural analysis, also appeared to be smaller in *Tff3-/-* mice on an HFD compared to WT mice (Figure 5). In females, the size of droplets was not altered, but the number of lipid droplets appeared to be increased in *Tff3-/-* females compared to WT females (Figure 5).

Lipid droplets were previously thought to be inert lipid reservoirs, but we now know that they are dynamic organelles that play a central role in many cellular functions, including a role in providing high-energy substrates used for fatty acid β-oxidation within mitochondria [56]. To analyse this further, we examined the liver fatty acid profile of *Tff3-/-* and WT animals (Table 2 and Table S3). Again, *Tff3*-/- mice of both sexes exposed to an HFD had significantly lower liver fat content when compared to WT mice (Table 2). The same was shown for control SD-fed mice (Table S3). Although not statistically significant, it is worth mentioning that HFD-treated females of both genotypes had lower liver lipid content compared to their male counterparts (Table 2). However, data from SD-fed mice show higher total liver fat content in females vs. males (Table S3), reinforcing the notion that females are more protected from liver fat accumulation upon HFD exposure [49,57,58]. The explanation for this dimorphism may lie in the oestrogen receptor α and its opposite regulation of lipid metabolism in male and female livers under dietary stress [49]. In addition, we analysed the fatty acid composition in the abdominal fat (Table S2) but could not detect any differences between the genotypes, suggesting that the liver could be the main site of impaired fatty acid metabolism regarding Tff3 role. 

Biochemical serum analyses revealed no significant differences, except for a significantly lower level of ALP in male Tff3-/- mice compared with WT male mice (Table 1). The lower level of ALP in *Tff3-/-* male animals demonstrates that Tff3 deficiency could be beneficial to liver function during prolonged HFD. Although without statistical significance, levels of serum total cholesterol and LDL were lower in *Tff3-/-* animals compared to WT mice (Table 1). 

In summary, histology, electron microscopy, fatty acid and blood serum analyses demonstrate a possible protective phenotype in *Tff3-/-* animals compared to WT mice on long-term HFD treatment. Moreover, *Tff3-/-* male mice gained less weight and showed better glucose utilization in glucose tolerance tests, especially at 21 weeks of age. When we compared *Tff3-/-* females with WT females, the differences became less obvious. No changes were observed in blood serum parameters (Table 1), weight gain (Figure 1) or glucose tolerance tests (Figure 2b and Figure 3b). However, *Tff3-/-* female mice also had ameliorated lipid accumulation in the liver (Figure 4d), and total fat content was reduced compared to WT female mice (Table 2). 

A previously published study on C57Bl/6J mice fed an HFD for 16 weeks showed that *Tff3* gene expression was downregulated in the liver of male mice and that restoration of Tff3 expression (adenovirus mediated overexpression) alleviated the fatty liver phenotype [15]. This observation was confirmed in two obesity and diabetes mouse models (Leprdb/Leprdb (db/db) and Leprob/Leprob (ob/ob) [13]. Moreover, the alleviation of the fatty liver phenotype was due to direct binding of Tff3 to *Pparα* and activation of fatty acid oxidation. Our data on the *Tff3-/-*/C57Bl6NCrl strain in the prolonged HFD model show opposite effects. *Tff3-*/- mice weigh less (Figure 1) and show better glucose utilization (Figure 2a) and lower lipid accumulation in the liver, as demonstrated by various methods (Figure 4 and Figure 5, Table 2), compared with WT mice when fed an HFD. Several factors could contribute to this contrary effect. Studies in which Tff3 was found to be a factor associated with improved diabetic phenotype [13,14,15] did not use a whole-body knockout but instead focused on liver Tff3 and restoration of Tff3, specifically in the liver. It appears that Tff3 deficiency in the whole organism (in the SD and HFD models) has protective effects with respect to metabolic health of the organism and reduces hepatic steatosis. We cannot exclude possible effects of Tff3 in organs other than the liver. Histological analyses performed on intestinal and perigonadal tissues from the same animals revealed no significant differences (data not shown). Another reason for this discrepancy is the contribution of additional genetic mutations present in a C57BL/6J substrain involved in metabolic regulation and affecting the metabolic phenotype [34,59]. In particular, loss of mitochondrial NAD (P)-transhydrogenase (Nnt) markedly exacerbates HFD-induced fatty liver disease in mice. HFD would increase mitochondrial dependence on NNT as a source of NADPH for antioxidant systems that counteract the development of NAFLD. Given the numerous genetic variances in the 6J strain, including loss of NNT function, these findings suggest that the 6N substrain is the most logical and representative genetic background model for metabolic studies [40]. 

Therefore, we intentionally generated a new *Tff3-/-* strain on a C57BL6/N genetic background. The inconsistencies in the current state of knowledge only confirm the complexity of this issue and highlight the need to continue systematic research to elucidate the role of Tff3 in complex metabolic processes in the liver.

To further evaluate the effect of Tff3 deficiency on Ppars signalling pathways and subsequent fatty acid oxidation processes, we analysed the gene expression of relevant markers in the liver. Although *Pparα* gene expression, previously associated with Tff3 [15], did not change between groups, peroxisome proliferator-activated receptor gamma (P*parγ*) was downregulated in both male and female *Tff3-/-* animals compared with WT (Figure 7a,b). We did not observe the same change in SD controls (Figure S6). In addition, we determined the amount of Pparγ protein in the liver tissues of male HFD mice and found the same trend of reduced Pparγ accumulation in male *Tff3-/-* mice but without statistical significance (Figure 8). *PPARγ* is a ligand-inducible transcription factor and belongs to the nuclear receptor superfamily, along with *PPARα* and *PPARδ/β* [60]. The Ppars family is known to modulate the expression of various genes that play key roles in lipid and glucose metabolism, making these proteins an important target for the treatment of diet-induced obesity and metabolic syndrome in general [61,62]. Under normal physiological conditions, *PPARγ* is predominantly expressed in adipose tissue. However, numerous studies have reported that HFD treatment led to an increase in *PPARγ* expression in liver tissue [63]. This upregulation in the liver activates downstream target genes involved in enhancing fatty acid uptake and synthesis, as well as lipid droplet formation. In other words, *Pparγ* has been shown to promote hepatic steatosis under the pathophysiological conditions of diet-induced obesity [64]. Our results show a reduced expression level of *Pparγ* in the absence of Tff3 protein, suggesting that reduced Ppary signalling contributes to reduced lipid accumulation in the liver. In addition, PPARγ is known to modulate the expression of genes involved in immune response [62]. 

Metabolic syndrome, including its hepatic manifestation (NAFLD), is marked by systemic low-grade inflammation triggered by nutrient overload. Progression of NAFLD to more severe forms due to chronic dietary exposure, e.g., nonalcoholic steatohepatitis (NASH), is characterized by an increased inflammatory state in the liver [65,66] The role of Tff3 in the immune response has been studied mainly in the context of gastrointestinal inflammatory pathologies, where it has been shown to elicit both pro- and anti-inflammatory activities [4]. Given the relevance of inflammation in the pathophysiology of fatty liver disease, as well as the involvement of Tff3 protein in immune signalling, we analysed the gene expressions of the relevant inflammatory markers in the livers of the studied animals (Figure 7a). The only significant change was an increased level of *Il-6* in *Tff3-/-* male mice compared to WT male mice. Western blot analysis showed upregulation of IL-6 in livers of *Tff3-/-* male mice compared to WT controls but without statistical evidence (Figure 8). Il-6 is a cytokine with a broad spectrum of physiological functions, including a role in pathophysiology of obesity and its associated states, although its exact role remains unknown [67]. *Il-6-/-* mice develop obesity, insulin resistance, hepatic steatosis and inflammation [68]. Even when fed an HFD, *Il-6-/-* mice showed a significantly worse fatty liver disease phenotype and insulin resistance compared with control animals. These results suggest that *Il-6* may be a relevant protective cytokine for diet-induced metabolic disorders. Interactions of *Tff3* and *IL-6* have been reported previously [69,70,71]. In response to bile duct injury, *IL-6* secretion is increased by activation of signal transducer activator of transcription 3 (*STAT3*) [70]. IL-6/STAT3 signalling initiates the expression of Tff3 in biliary epithelial cells (BEC) to promote proliferation and migration and facilitate wound healing. In the present study, the significant upregulation of *Il-6* in the liver of *Tff3*-/- male mice may explain the observed partial protection and reduced fatty liver phenotype in the absence of Tff3. *Il-6* was also upregulated in the livers of female WT mice compared with male WT mice (Figure 7c), which fits the hypothesis that upregulation of *Il-6* in the liver is a protective factor in response to HFD-induced metabolic syndrome. Interestingly, *Tff3-/-* female mice showed a different inflammatory gene expression profile compared with *Tff3-/-* male mice (Figure 7d). *Il-6* did not change, but C*d68*, *Tnfα* and *Tgfβ* were upregulated. Moreover, *Tff3-/-* female mice showed significant downregulation of *Cxcl1* and *Ccr2* when compared to WT female mice (Figure 7b). 

It is important to emphasize that in each experiment we performed, the sex of the animals (both in WT and *Tff3-/-* mice) was an important variable leading to different results, which speaks to the relevance of including both sexes in biomedical research and the need to clearly indicate which strain and sex of animal models are being used. In molecular analyses of the liver tissue described above, we focused on *Tff3-/-* male mice to a greater extent because these animals showed more prominent changes in overall metabolic status and histological liver examinations. Nevertheless, *Tff3-/-* female mice clearly exhibit different responses to HFD treatment compared to WT females, and the underlying mechanisms are not the same as those in males with Tff3 deficiency. These results raise new questions that should be investigated in future research.

In conclusion, when *Tff3-/-* mice and WT mice of both sexes were fed an HFD for 8 months, *Tff3-/-* male mice, upon 21 weeks of HFD exposure, had lower body weights and better glucose utilization. Prolonged exposure (32 weeks) to an HFD resulted in no differences in body weight nor functional metabolic tests. Tff3-deficient mice had ameliorated fatty liver phenotype, as evidenced by significantly reduced lipid droplet content in hepatocytes, as well as decreased total liver fat content compared to male WT mice. In addition, at the molecular level, we detected significantly changed expression of the *Pparγ* (downregulation) and *Il-6* (upregulation) genes, both of which are involved in the regulation of lipid metabolism and the pathogenesis of hepatic steatosis. Protein levels showed the same trend but without statistical significance due to higher individual variance. Our study showed, for the first time, differential effects of Tff3 deficiency in male and female mice and specific dynamics of the effects. The difference in the phenotype of Tff3 -/-/C57Bl6N compared with other polygenic diabesity mouse models points to the often-underappreciated impact of additional gene defects (such as the Nnt mutation present in C57BL6J) and interactions in animal models of complex diseases. 

Overall, the involvement of *Tff3* in regulation of lipid metabolism identified it as a possible candidate for treatment of hepatic manifestations of metabolic syndrome. Our results provide new insights and a basis for future research directions into a possible mechanistic explanation for the observed phenotype; therefore, our newly generated congenic *Tff3-/-/*C57Bl6N mouse strain represents a valuable tool in this scientific pursuit. 

## Figures and Tables

**Table 1:** Biochemical analysis of blood sera.

Parameter	Unit	Group				
		WT ♂	*Tff3-/-* ♂	WT ♀	*Tff3-/-* ♀	*Genotype × Sex*
**LDL**	mmol/L	3.19 ± 0.92	2.18 ± 0.49	1.54 ± 0.38	1.01 ± 0.27	0.304
**HDL**	mmol/L	2.17 ± 0.18	1.91 ± 0.17	1.29 ± 0.15	1.30 ± 0.20	0.058
**Total cholesterol**	mmol/L	5.74 ± 1.10	4.40 ± 0.69	3.18 ± 0.36	2.60 ± 0.43	0.178
**Triglycerides**	mmol/L	0.86 ± 0.16	0.67 ± 0.12	0.77 ± 0.12	0.65 ± 0.15	0.519
**BG**	mmol/L	14.7 ± 4.12	16.9 ± 3.88	14.5 ± 1.26	12.6 ± 3.69	0.135
**AST**	U/L	366.9 ± 187.9	304.0 ± 148.2	524.7 ± 320.8	378.4 ± 212.7	0.611
**ALT**	U/L	216.1 ± 115.1	83.2 ± 44.1	137.2 ± 114.6	61.6 ± 47.0	0.389
**CRP**	mg/L	0.16 ± 0.02	0.13 ± 0.02	0.13 ± 0.03	0.13 ± 0.02	0.255
**Urea**	mmol/L	9.17 ± 2.04	9.45 ± 1.32	8.07 ± 1.10	8.38 ± 1.09	0.978
**ALP**	U/L	81.8 ± 29.4	51.0 ± 5.3	67.3 ± 4.6	67.7 ± 12.6	0.031 *
**Total protein**	g/L	54.3 ± 17.8	54.2 ± 4.07	58.0 ± 6.1	50.9 ± 3.6	0.395

Data are presented as mean ± SD and were compared by two-way ANOVA, followed by Bonferroni post hoc test; *p* ≤ 0.05 was considered statistically significant (*p* values for the effect of genotype × sex; statistically significant values are marked in bold letters). Significant changes in sex × genotype interaction were determined by appropriate post hoc tests and are marked as follows: * WT ♂ vs. *Tff3-/-* ♂. Description: LDL—low-density lipoprotein, HDL—high-density lipoprotein, BG—blood glucose, AST—aspartate aminotransferase, ALT—alanine aminotransferase, CRP—C-reactive protein, ALP—alkaline phosphatase. ♂-male; ♀-female.

## Supplementary Materials

The following supporting information can be downloaded at: https://www.mdpi.com/article/10.3390/life12081288/s1, Table S1. Oligonucleotides used for qPCR analysis; Table S2. Fatty acid (average) content of abdominal fat in mice on high fat diet; Figure S1. Masson-Goldner staining of livers from male and female WT and *Tff3-/-* mice on HFD; Figure S2. Effect of long-term treatment with HFD and effect of gender on the expression of Tff3 in the liver tissue of WT mice; Figure S3. Intraperitoneal glucose tolerance (GTT) performed on 21 (A)-and 36 (B) week-old WT and *Tff3-/-* mice (male and female) on standard diet (SD); Figure S4. Intraperitoneal insuline tolerance (ITT) test performed on 21 (A)-and 36 (B) week-old WT and *Tff3-/-* mice (male and female) on standard diet (SD); Figure S5. Histologic evaluation of livers from standard diet (SD) male and female WT and Tff3-/- mice; Table S3. Fat (average ± standard error) and Fatty acid content (average) of liver mice on standard diet; Figure S6. Effect of Tff3 deficiency on Pparγ, Il-6, Cxcl1 and Ccr2 gene expression in liver of SD-fed mice.

## Abbreviations

*Actβ*	actin beta
ALT	alanin–aminotransferase
ALP	alkaline phoshatase
AST	aspartate aminotransferase
*β2m*	beta-2-microglobulin
BG	blood glucose
*Ccr2*	chemokine (C-C motif) receptor 2
*Cd68*	mouse CD68 antigen
*Cgl58*	abhydrolase domain containing 5
*Chreb*	carbohydrate response element binding protein
*Cpt1a*	carnitine palmitoyltransferase I
CRP	C-reactive protein
*Cxcl1*	C-X-C motif chemokine ligand 1
*Cxcr7*	atypical chemokine receptor 3
*Cyp21*	cytochrome P450, family 21, subfamily a, polypeptide 1
*Dgat1*	diacylglycerol O-acyltransferase 1
*Fabp1*	fatty acid binding protein 1
*Fitm2*	fat storage-inducing transmembrane protein 2
GI	gastrointestinal
*GlyK*	glycerol kinase
HDL	high-density lipoprotein
HE	hematoxylin and eosin
HFD	high-fat diet
*Hmgcs2*	hydroxymethylglutaryl-CoA synthase
*Il-1α*	interleukin 1 alpha
*Il-1β*	interleukin 1 beta
*Il-6*	interleukin 6
*Il-14*	interleukin 14
*Irs1*	insulin receptor substrate 1
*Irs2*	insulin receptor substrate 2
IPGTT	intraperitoneal glucose tolerance tests
IPITT	intraperitoneal insulin tolerance tests
*Mcp1*	monocyte chemoattractant protein-1
MUFA	monounsaturated fatty acids
NAFLD	nonalcoholic fatty liver disease
*Pgc1α*	Pparγ coactivator
*Ppar α*	peroxisome proliferator activated receptor alpha
*Ppar γ*	peroxisome proliferator activated receptor gamma
PUFA	polyunsaturated fatty acids
qPCR	quantitative polymerase chain reaction
SD	standard diet
SFA	saturated fatty acids
*Srebf1*	sterol regulatory element binding transcription factor 1
*Stat3*	signal transducer activator of transcription 3
T1D	type 1 diabetes
T2D	type 2 diabetes
Tffs	trefoil factor family proteins
Tff3	trefoil factor family 3
*Tgfβ*	tumor growth factor beta
*Tnfα*	tumor necrosis factor alpha
WT	Wild type
