# Supplementary material for: Tff3 Deficiency Protects against Hepatic Fat Accumulation after Prolonged High-Fat Diet
Source: Life (Basel). 2022 Aug 22;12(8):1288. doi: 10.3390/life12081288 (PMC9409972; doi:10.3390/life12081288)
Supplement: Supplementary file 1 [file life-12-01288-s001.zip › life-1742338-supplementary.pdf]

Supplementary data

Table S1. Oligonucleotides used for qPCR analysis.

Gene Symbol	Accession No.	Primer Sequence Forward (5′-3′) Reverse (5′-3′)	Optimized qPCR Conditions (Annealing Temp/MgCl)
		**Cytokines**	
Ccr2	NM_009915.2	GGTCTGGTTGGGTTGTAAA<br>GTCTTTGAGGCTTGTTGCTATG	59°C; 3 mM
Cd68	NM_001291058.1	CTCTTGCTGCCTCTCATCATT<br>CTGGTAGGTTGATTGTCGTCTG	58 °C; 2,5 mM
Cxcl1	NM_008176.3	GTGTCAACCACTGTGCTAGT<br>CACACATGTCCTCACCCTAATAC	61 °C; 3.5 mM
Cxcr7	NM_001271607.1	GACCATGTAGGCCTCAGATTAG<br>CAGCCGAGACTGGCATAAA	63 °C; 3.5 mM
Il-1α	NM_010554.4	CCTTACACCTACCAGAGTGATTT<br>CCTTACACCTACCAGAGTGATTT	65 °C; 3 mM
Il-1β	NM_008361.4	ATGGGCAACCACTTACCTATTT<br>GTTCTAGAGAGTGCTGCCTAATG	64 °C; 3 mM
Il-6	NM_031168.2	GATAAGCTGGAGTCACAGAAGG<br>TTGCCGAGTAGATCTCAAAGTG	59 °C; 3.5 mM
Il-14	NM_001005506.3	CCTCACTTCAGCTACCTCTTAAA<br>CTACAAGTGGATGGAGGGAAAG	61 °C; 3.5 mM
Mcp1	NM_011333.3	CCTGGATCGGAACCAAATGA<br>CGGGTCAACTTCACATTCAAAG	62 °C; 3 mM
Tgfα	NM_031199.4	CTTTAGGAAGGACCTGGGTTG<br>GTGTGTCCAGGCTCCAAATA	66 °C; 3 mM
Tnfα	NM_013693.3	GTCTCAGAATGAGGCTGGATAAG<br>CATTGCACCTCAGGGAAGAA	63 °C; 2.5 mM
		**Fatty Acid Metabolism**	
Cgl58	NM_026179.2	ATGCTGTGGAATGAGGACATAG<br>CATAGTGAGTGGCTGGTGAAA	59°C; 2,5mM
Chreb	NM_021455.5	CAGCTGCGGGATGAAATAGA<br>CAAAGCGCTGATGTGTGATG	61°C; 2,5mM
Cpt1α	NM_013495.2	TCGAAACCCAGTGCCTTAAC<br>AAGCAGCACCCTCACATATC	58°C 2,5 mM
Cyp21	NM_009995.2	CTGGGTCGGAGCTTCATTT<br>GTCTTGACTCTCTTCCCTTGAC	59°C; 3,5mM
Dgat1	NM_010046.3	CCAACCATCTGATCTGGCTTAT<br>GACTCAGCATTCCACCAATCT	65°C; 3mM
Glyk	BC003767.1	GCACTAGAAGCTGTTTGTTTCC<br>GCTGGTCATTCCTCCATCTAC	58°C; 2,5 mM
Hmgcs2	NM_008256.4	CCTGTGAAGAGGGAGATGAAAG<br>GCCCACAGTCTGAGAATAAGC	64°C; 3 mM
Fabp1	NM_017399.5	AAGTCAAGGCAGTCGTCAAG<br>TGGTATTGGTGATTGTGTCTCC	59°C; 3,5mM
Fitm2	NM_173397.4	GACAGGAGGACAATGGCTAAT<br>CCACACCAAAGGTACCTAGTAAG	56°C; 2,5mM
Irs1	NM_010570.4	GTCAGGGACACTCTTGACTAAC<br>TGCCAAGGAAAGACAGGATAAA	61°C; 2,5mM
Irs2	NM_001081212.2	CTGCTGCTCACTTTCCTATCA<br>CCTGCCTCTTGGTTCCTTATC	61°C; 2,5mM
Pgc1α	NM_001127330.2	GCCTAAGTTTGAGTTTGCTGTG<br>GCGGTCTCCACTGAGAATAATG	58°C 2,5 mM
Pparα	NM_011144.6	GCTCGTACAGGTCATCAAGAAG<br>CTGCCATCTCAGGAAAGATCAG	59°C 2,5 mM

Pparγ	NM_0011273 30.2	GCCTAAGTTTGAGTTTGCTGTG GCGGTCTCCACTGAGAATAATG	59°C 2,5 mM
Srebpf1	NM_011480. 4	AGCCCTCCACCAGGTAATAA GGGTTCCCAGTCTACTCACTAA	61°C; 2,5mM
Reference (housekeeping) genes			
Actβ	NM_007393. 5	GCAAGCAGGAGTACGATGAG CCATGCCAATGTTGTCTCTT	61 °C; 3.5mM
β2m	NM_009735. 3	CCTGCAGAGTTAAGCATGACAGT TCATGATGCTTGATCACATGTCT	60 °C; 3 mM

Ccr2- chemokine (C-C motif) receptor 2; *Cd68*- mouse CD68 antigen; *Cgl58*- abhydrolase domain containing 5; *Chreb*-carbohydrate response element binding protein; *Cpt1α*- carnitine palmitoyltransferase I; *Cxcl1*- C-X-C motif chemokine ligand 1; *Cxcr7*- atypical chemokine receptor 3; *Cyp21*- cytochrome P450, family 21, subfamily a, polypeptide 1; *Dgat1*- diacylglycerol O-acyltransferase 1; *Fabp1*-fatty acid binding protein 1; *Fitm2*- fat storage-inducing transmembrane protein 2; *Glyk*- glycerol kinase; *Hgmcs2*- hydroxymethylglutaryl-CoA synthase; *Il-1α*- interleukin 1 alpha; *Il-1β*- interleukin 1 beta; *Il-6*- interleukin 6; *Il-14*-interleukin 14; *Irs1*- insulin receptor substrate 1; *Irs2*- insulin receptor substrate 2; *Mcp1* - monocyte chemoattractant protein-1; *Srebf1*- sterol regulatory element binding transcription factor 1; *Tgfβ*- tumor growth factor beta; *Tnfα*- tumor necrosis factor alpha; *Pgc1α*- Pparγ coactivator; *Pparα* - peroxisome proliferator activated receptor alpha VI; *Ppar γ*-peroxisome proliferator activated receptor gamma; *Actβ* – actin beta; *β2m* – beta 2 microglobulin

Table S2. Fatty acid (average) content of abdominal fat in mice on high fat diet.

**Main fatty acids (g of fatty acids/100 g of total fatty acids**	**Groups**			
	**WT ♂**	**Tff3-/- ♂**	**WT ♀**	**Tff3-/- ♀**
C 14:0	0.77 *	0.78 †	0.99	1.10
C 16:0	19.01 *	18.45 †	20.06	21.44
C 16:1	5.85	5.90	6.33	5.85
C 18:0	3.76	3.20 †	4.17	4.63
C 18:1	54.77	54.97 †	52.17	50.02
C 18:2, n-6	13.19	14.06	13.12	13.61
C 18:3, n-3	0.48	0.48 †	0.59	0.64
C 20:1, n-9	0.51	0.53	0.49	0.53
C 20:2, n-6	0.18 *	0.19 †	0.25	0.27
C 20:4, n-6	0.25 *	0.21 †	0.36	0.37
C 22:6, n-3	0.09 *	0.08 †	0.17	0.18
∑ SFA [1]	24.03	22.87 †	25.72	27.68
∑ MUFA [2]	61.45	61.74 †	59.33	56.73
∑ PUFA [3]	14.47 ‡	15.33	14.89	15.50
n-6/n-3 PUFA [4]	21.30 : 1 *	22.56 : 1 †	15.88 : 1	15.09 : 1

[1] Saturated fatty acids. [2] Monounsaturated fatty acids. [3] Polyunsaturated fatty acids. [4] Ratio of omega -6 and omega -3 polyunsaturated fatty acids. Results are presented as mean and were analysed using general linear models (GLM) procedures of the SAS/STAT module (SAS Institute Inc., Cary, NC, USA), the differences being determined by a Tukey–Kramer multiple comparison test, taking into consideration the genotype as the main effect, separately for male and female mice. Statistical significance was considered at $p < 0.05$. *—WT ♂ vs WT ♀ (sex related diff.); † - Tff3-/-o ♂ vs Tff3-/- ♀ (sex related diff.); ‡ - WT ♂ vs Tff3-/- ♂ (gene related diff.).

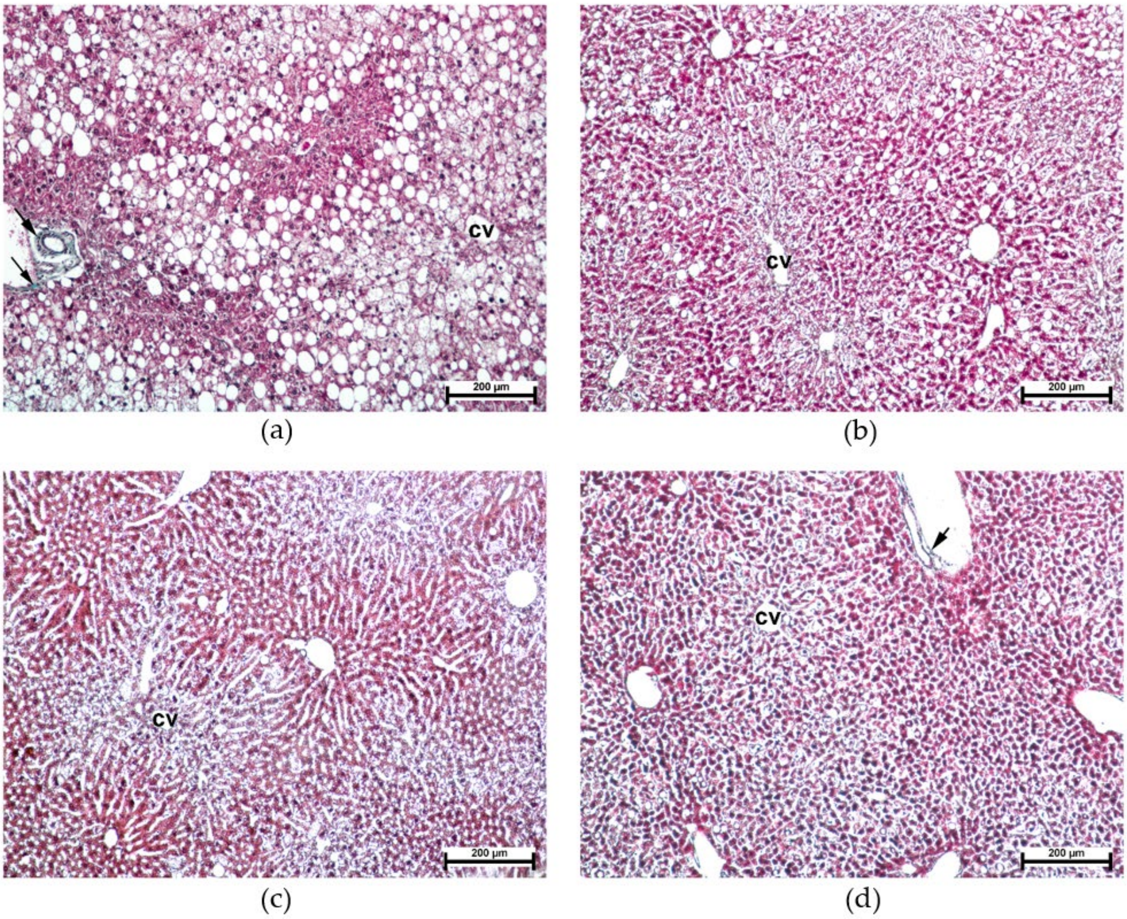

Figure S1. Masson-Goldner staining of livers from male and female WT and *Tff3*-/- mice on HFD, (**a**) WT male (**b**) WT female (**c**) *Tff3*-/- male (**d**) *Tff3*-/-female. Collagen fibers (green; arrows); cv, central vein. Scale bars 200 µm.

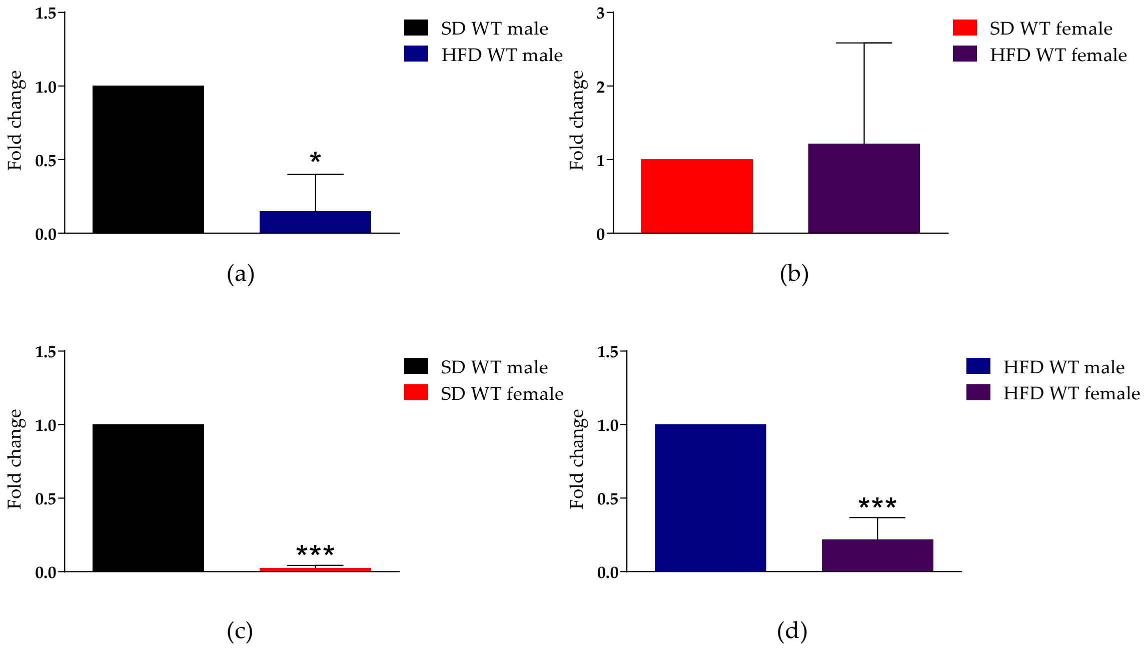

Figure S2. Effect of long-term treatment with HFD and effect of gender on the expression of Tff3 in the liver tissue of WT mice. We performed qPCR for all animal groups (n = 5) using the SYBR green detection system. Ct values were analyzed using REST© software and results are expressed as fold change and SEM. *Tff3* gene expression of WT male fed with long term HFD compared to age matched (9-month-old) WT male fed with SD (**a**) WT female HFD compared to WT female SD (**b**) WT female fed with SD compared to WT male SD (**c**) and WT female fed with HFD compared to WT male fed with HFD (**d**). Statistically significant time points marked as * $p \leq 0.05$; *** $p \leq 0.001$.

(A) **GTT 21-week-old Standard diet**

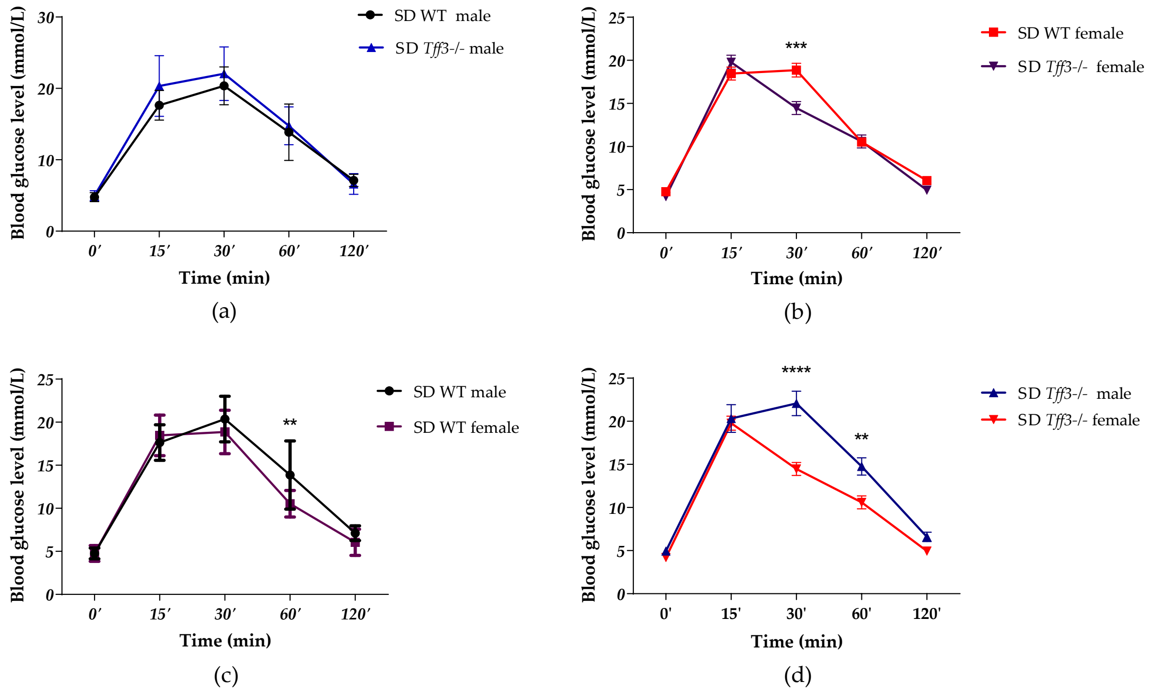

(**B**) **36 weeks old Standard diet**

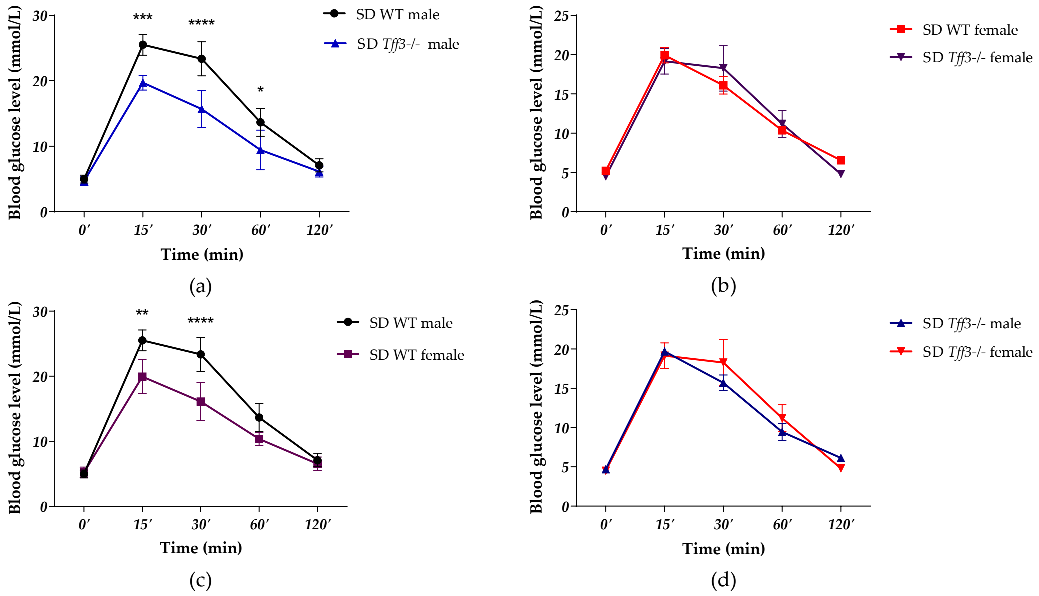

Figure S3. **Intraperitoneal glucose tolerance (GTT) performed on 21 (A)-and 36 (B) week-old WT and *Tff3*-/- mice (male and female) on standard diet (SD).** Blood glucose levels were measured at time points 0, 15, 30, 60 and 120 min after glucose injection (2 mg/g body mass) and showed as (**a**) WT male compared to *Tff3*-/- male, (**b**) WT female compared to *Tff3*-/- female (**c**) WT male compared to WT female and (**d**) *Tff3*-/- male compared to *Tff3*-/- female; 2 way ANOVA (Tukey post hoc test) was used for statistical analysis and significant time points are marked as * $p \leq 0.05$; ** $p \leq 0.01$; *** $p \leq 0.001$; **** $p \leq 0.0001$

(**A**) **ITT 23-week-old Standard diet**

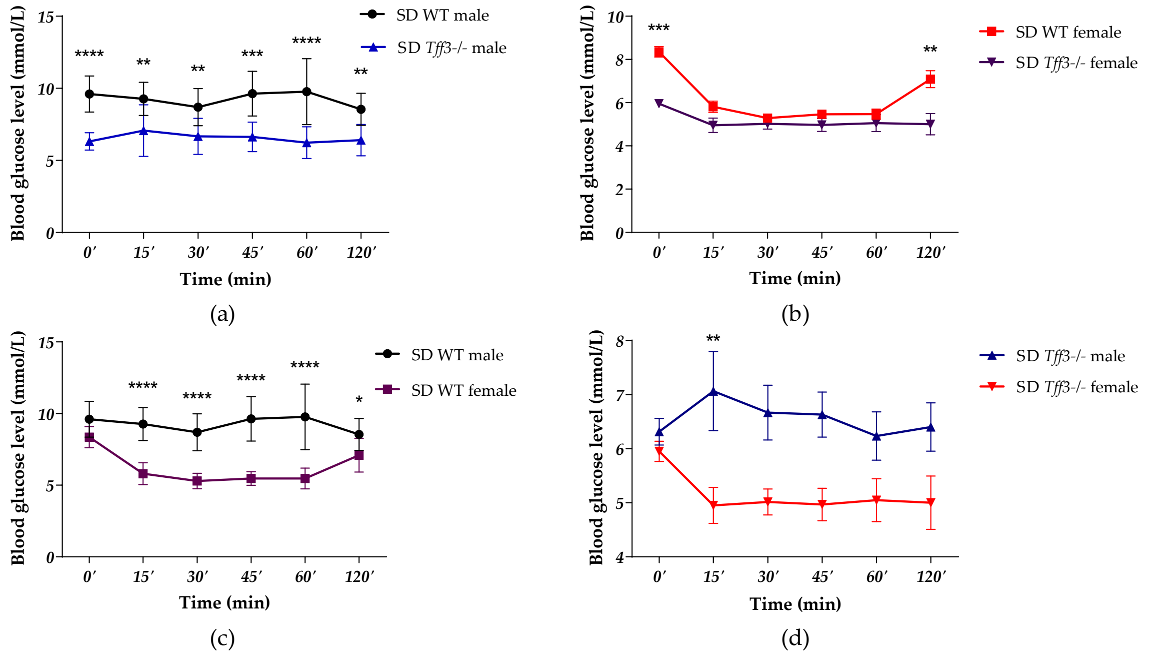

(**B**) **ITT 38-week-old Standard diet**

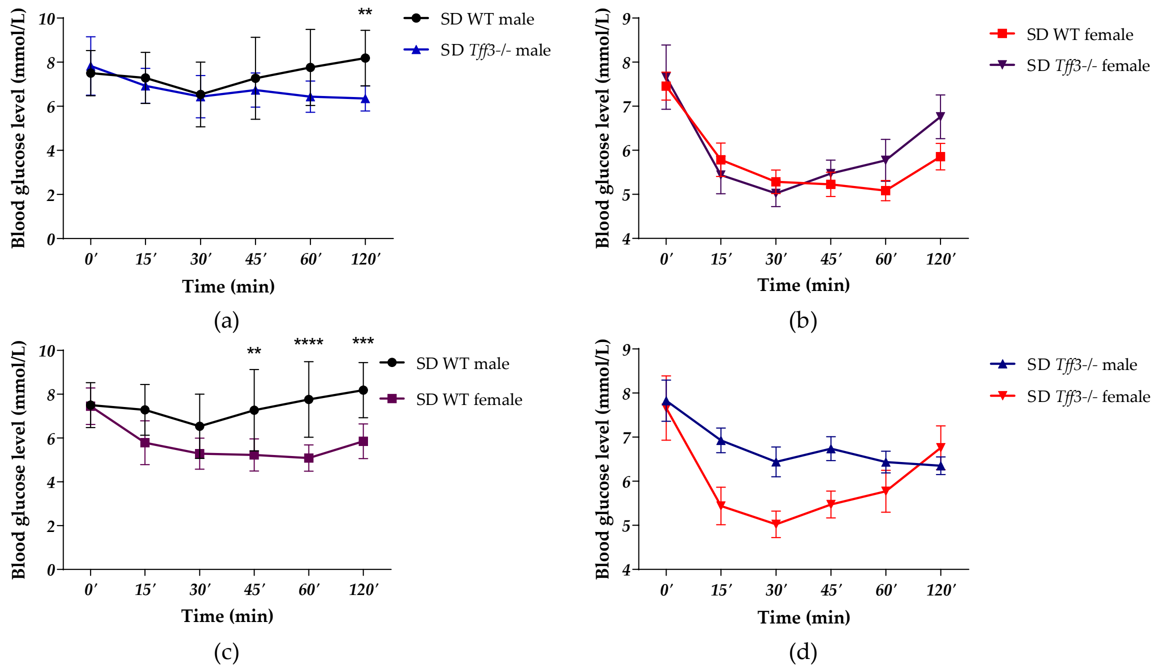

Figure S4 . Intraperitoneal insuline tolerance (ITT) test performed on 21 (A)-and 36 (B) week-old WT and *Tff3*-/- mice (male and female) on standard diet (SD). Blood glucose levels were measured at time points 0, 15, 30, 60 and 120 min after glucose injection (2 mg/g body mass) and showed as (**a**) WT male compared to *Tff3*-/- male, (**b**) WT female compared to *Tff3*-/- female (**c**) WT male compared to WT female and (**d**) *Tff3*-/- male compared to *Tff3*-/- female. Tff3 deficent mice at age of 23 weeks fasting for 4h have lower starting blood glucose level (Fig.S4A). Glucose level after longer fasting hours 16h (GTT) is not affected by Tff3 deficiency. 2 way ANOVA (Tukey post hoc test) was used for statistical analysis and significant time points are marked as * $p \leqslant 0.05$; ** $p \leqslant 0.01$; ***$p \leqslant 0.001$; **** $p \leqslant 0.0001$.

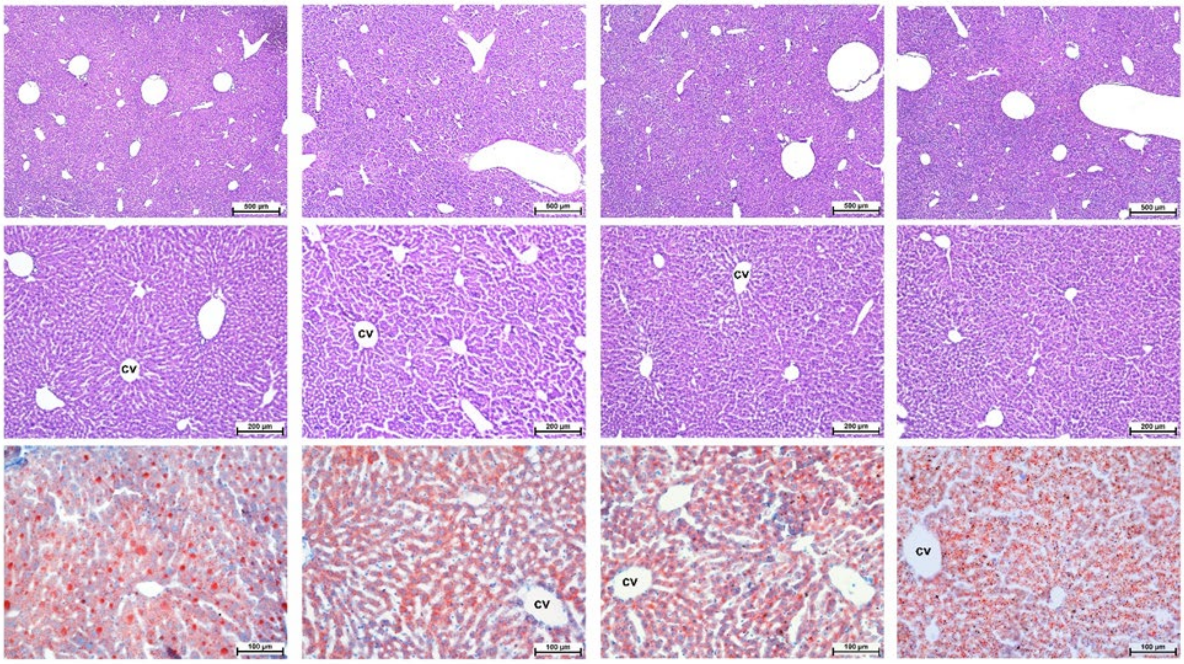

(**a**) (**b**) (**c**) (**d**)

Figure S5. Histologic evaluation of livers from standard diet (SD) male and female WT and Tff3-/- mice, (**a**) WT male (**b**) Tff3-/- male (**c**) WT female (**d**) Tff3-/-female. Representative liver sections were stained with HE (upper and middle row panels) and Oil Red O (bottom row panels). Scale bars 500 μm (upper row panels), 200 μm (middle) and 100 μm (bottom row panels). HE- hematoxylin eosin, cv-central vein.

Table S3. Fat (average ± standard error) and Fatty acid content (average) of liver mice on standard diet.

**Main fatty acids (g of fatty acids/100 g of total fatty acids**	**WT ♂**	**Tff3-/- ♂**	**WT ♀**	**Tff3-/- ♀**
C 14:0	0.60	0.55	0.74	0.66
C 16:0	22.94 ‡	25.43	22.71 §	24.27
C 16:1	7.28	5.88	5.74	5.44
C 18:0	3.68	5.46	3.84	5.59
C 18:1	25.68 ‡*	21.15†	31.55 §	28.18
C 18:2, n-6	23.35	21.56	21.81	18.40
C 18:3, n-6	0.51	0.42	0.71	0.56
C 18:3, n-3	1.06	0.94	0.92	0.74
C 20:1, n-9	0.33 *	0.28†	0.24	0.22
C 20:3, n-6	0.58 *	0.72	0.35	0.58
C 20:4, n-6	4.85	6.88	4.05	5.88
C 20:5, n-3	0.77 *	0.80	0.49	0.71
C 22:4, n-6	0.16	0.20	0.16	0.19
C 22:5, n-6	0.09	0.11	0.08	0.08
C 22:5, n-3	0.61 *	0.67	0.39	0.50
C 22:6, n-3	6.27 *	7.72	5.02	6.89
∑ SFA [1]	28.03 ‡	32.26	28.09 §	31.24
∑ MUFA [2]	33.35 ‡	27.37	37.58 §	33.90
∑ PUFA [3]	38.46 *	40.17 †	34.16	34.67
n-6 PUFA	29.18	29.62 †	28.56	25.27
n-3 PUFA	8.89	10.28	7.00	9.00
n-6/n-3 PUFA	3.39 : 1	2.92 : 1	3.80 : 1	2.91 : 1
Fat content (g/100 g liver)	15.0 ± 1.72 ‡*	9.9 ± 1.88	19.7 ± 1.72 §	13.9 ± 1.88

[1] Saturated fatty acids. [2] Monounsaturated fatty acids. [3] Polyunsaturated fatty acids. Results are presented as mean and as mean±*standard error (for fat content) and were* analysed using general linear models (GLM) procedures of the SAS/STAT module (SAS Institute Inc., Cary, NC, USA), the differences being determined by a Tukey–Kramer multiple comparison test, taking into consideration the genotype as the main effect, separately for male and female mice. Statistical significance was considered at $p \leq 0.05$. *—WT ♂ vs WT ♀ (sex related diff.); † - Tff3-/- ♂ vs Tff3-/- ♀ (sex related diff.); ‡ - WT ♂ vs Tff3-/- ♂ (gene related diff.); § - WT ♀ vs Tff3-/- ♀ (gene related diff.)

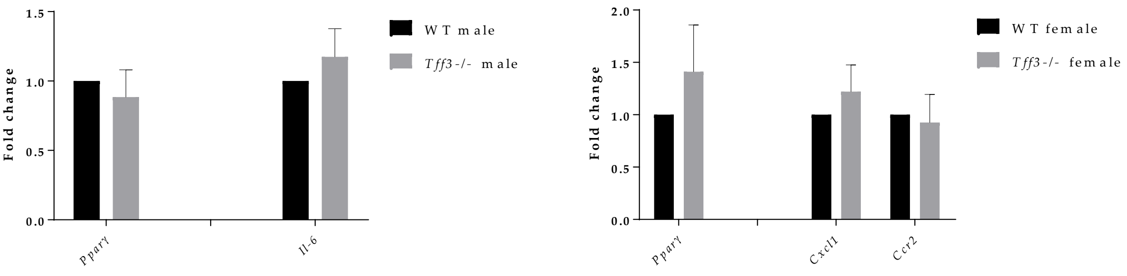

(a) (b)

Figure S6. Effect of Tff3 deficiency on Pparγ, Il-6, Cxcl1 and Ccr2 gene expression in liver of SD-fed mice. We performed qPCR using the SYBR green detection system. Ct values were analyzed using REST© software and results are expressed as fold change. (**a**) *Pparγ* and *Il-6* gene expression in liver of *Tff3-/-* male mice compared to WT male mice (both fed with SD) (**b**) *Pparγ*, *Cxcl1, Ccr2* gene expression in liver of *Tff3-/-* female mice compared to WT female mice (both fed with SD)
